# Molecular profile in Paraguayan colorectal cancer patients, towards to a precision medicine strategy

**DOI:** 10.1002/cam4.2191

**Published:** 2019-05-06

**Authors:** Tania Fleitas-Kanonnikoff, Carolina Martinez‐Ciarpaglini, Josefina Ayala, Cinthia Gauna, Rita Denis, Ita Yoffe, Silvia Sforza, María Teresa Martínez, Alicia Pomata, Maider Ibarrola‐Villava, Sipan Arevshatyan, Verónica Burriel, Diego Boscá, Oscar Pastor, Ana Ferrer‐Martinez, Francisca Carrasco, Cristina Mongort, Samuel Navarro, Gloria Ribas, Andres Cervantes

**Affiliations:** ^1^ Department of Medical Oncology CIBERONC Biomedical Research Institute INCLIVA University of Valencia Valencia Spain; ^2^ Department of Pathology CIBERONC Biomedical Research Institute INCLIVA University of Valencia Valencia Spain; ^3^ Department of Medical Oncology Instituto Nacional del Cáncer (INCAN) Capiatá Paraguay; ^4^ Department of Medical Oncology Hospital de Clínicas (HC) Universidad Nacional de Asunción San Lorenzo Paraguay; ^5^ Laboratorios Curie SA Asunción Paraguay; ^6^ Department of Pathology Instituto Nacional del Cáncer (INCAN) Capiatá Paraguay; ^7^ Gembiosoft‐Universidad Politécnica de Valencia Valencia Spain; ^8^ Veratech Valencia Spain

**Keywords:** colorectal cancer, microsatellite instability, mutational profile, Oncocarta, precision medicine

## Abstract

Somatic mutation analysis and evaluation of microsatellite instability (MSI) have become mandatory for selecting personalized therapy strategies for advanced colorectal cancer and are not available as routine methods in Paraguay. The aims of this study were to analyze the molecular profile as well as the microsatellite status in a series of advanced colorectal patients from two public hospitals from Paraguay, to introduce these methodologies in the routine practice to guide the therapeutic decisions. Thirty‐six patients diagnosed with advanced colorectal cancer from two referent public hospitals from Paraguay were recruited from May 2017 to February 2018. Sequenom Mass spectrometry, Oncocarta Panel V.1 was applied to analyze the mutational profile from formalin‐fixed paraffin‐embedded samples. The microsatellite status was tested by immunohistochemistry (IHC). The mean age of the patients was 52 years with a range from 20 to 74 years. Eighty‐three percent of the patients included in the study have advanced‐stage tumors at the moment of the diagnosis. Sixteen patients (44.4%) were wild‐type for all the oncogene regions analyzed with the Oncocarta panel. Thirty‐two hot‐spot pathogenic variants on seven oncogenes, among 20 patients (55.6%), were identified, including KRAS, NRAS, BRAF, PI3KCA, FGFR, epidermal growth factor receptor, and PDGFRA. Moreover, 14 (38.8%) of these patients presented pathogenic variants in KRAS/NRAS or BRAF genes that have implications in the clinical practice decisions. Five patients (14%) presented MSI. The IHC study for microsatellite status and the molecular profile analysis through Sequenom mass spectrometry are feasible and useful methods, due to identify those patient candidates for targeted therapies and for the budgetary calculations of the National Health Plans.

## 1 INTRODUCTION

Colorectal cancer (CRC) is the third most common cancer worldwide in terms of incidence, but also the second in terms of mortality with over 1.8 million new CRC cases and 881 000 deaths estimated to occur in 2018.^1^ The highest CRC incidence rates are found in some parts of Europe (including Hungary, Slovenia, Slovakia, the Netherlands, and Norway), Australia/New Zealand, Northern America, and Eastern Asia. As an extent of westernization, CRC now ranks as the top five cancers in Latin‐America, being the second and third leading cause of cancer death in South America and the Caribbean, respectively.^2^ Paraguay occupies the 19th position within Latin‐American countries with an incidence of 15 and 12.7 per 100.000 in men and women, respectively, and a mortality of 9 and 7.5 per 100.000 in men and women, respectively.^1^

CRC in Latin‐American countries is diagnosed at an advanced stage of the disease in almost 80% of cases, especially in those related with low socioeconomic conditions.^3^ Metastatic disease initially is not suitable for potentially curative resection. Therefore, advanced target therapies, such as monoclonal antibodies (bevacizumab) or proteins (aflibercept) against vascular endothelial growth factor, and against the epidermal growth factor receptor (EGFR), respectively, in combination with chemotherapy, should be considered in patients with metastatic CRC (mCRC), since they improve the outcome of mCRC patients.^4^, ^5^, ^6^, ^7^ However, the high costs associated with these targeted therapies limit their application in developing countries, including Paraguay.^8^, ^9^

The gold standard of the therapeutic strategy includes a multidisciplinary management and an early approach to the disease. For the advanced disease, chemotherapy associated with targeted therapies selected according to the pathological and molecular profile of the tumor, increases the median overall survival (OS) to around 30 months. Factors which may have contributed to this improvement in the OS include: (a) continuous and more exhaustive follow‐up, (b) improvements in the efficacy of systemic therapies, (c) inclusion of biomarker‐based patient selection.^10^

Somatic mutation analysis has become mandatory for selecting personalized therapies for CRC. Mutation profiling of the *RAS*/*BRAF* pathway could guide the selection of patients with potential benefit from anti‐EGFR therapies.^5^, ^11^, ^12^ Pathogenic variants in *KRAS* or *NRAS* (expanded *RAS* analysis) predict a lack of response to EGFR‐targeting monoclonal antibodies. Moreover, this targeted therapy has a detrimental effect in patients with *RAS*‐mutant tumors, specifically when combined with an oxaliplatin‐based cytotoxic backbone.^13^, ^14^, ^15^ *BRAF* pathogenic variants (mainly V600E) are found in around 8%‐12% of patients with mCRC included in clinical trials and are almost exclusively nonoverlapping with other *RAS* pathogenic variants. *BRAF* pathogenic variants are a significant negative prognostic marker for patients with mCRC.^16^ Moreover, two meta‐analyses demonstrated that the benefit of EGFR antibody therapies was greater in patients with *RAS* wild‐type/*BRAF* wild‐type tumors than in those with *RAS* wild‐type/*BRAF*‐mutant tumors.^11^, ^17^ Methods for molecular testing include Sanger sequencing, pyrosequencing, next‐generation sequencing (NGS) technology, and mass spectrometry with different spectrum of advantages/disadvantages.^18^ The mass spectrometry technique, matrix‐assisted laser desorption/ionization‐time of flight, is a cost‐effective method that has been used to assess point mutations across different solid tumors.^19^, ^20^ The Sequenom MassARRAY technology, in combination with a commercial kit called OncoCarta v1.0, is a commercial panel that screens 238 somatic pathogenic variants across 19 oncogenes exploring somatic changes in oncogenes with known responses or resistance‐targeted therapy in solid tumors. This methodology was applied to our series of patients diagnosed with CRC in order to explore the actionable mutational profile that could guide the clinical decisions.

The evaluation of microsatellite instability (MSI) in CRC through the immunohistochemistry (IHC) study for mismatch repair proteins (MMR) expression has become mandatory in daily practice for various reasons. MMR deficiency is the main characteristic of the CMS1 group of the latest CRC consensus molecular classification.^21^ This group of tumors is linked to specific clinicopathologic features with lower rates of response to chemotherapy and shorter disease‐free survival periods after treatment. About 15% of CRC arise through the MSI pathway and most of these tumors are sporadic.^22^ However, the IHC study for MMR proteins is recommended for the detection of the hereditary nonpolyposis CRC syndrome (Lynch syndrome) accounting for 1% to 5% of all the cases.^23^ Moreover, MSI is the only predictive biomarker approved by the FDA for the immune checkpoint blockade inhibitors therapy with pembrolizumab for solid tumors and nivolumab in CRC. MMR‐deficient mCRC, represents approximately 4% of all mCRC cases and is characterized by very high levels of mutations. Extensive basic research and clinical trial efforts are underway to identify the optimal therapy combinations that are needed for this CRC subset.^24^, ^25^

*HER2* amplification is a relevant potential target in CRC. Recent studies of *ERBB2* amplification and sequence pathogenic variants in CRC suggest that *HER2* is a therapy target in this disease, in addition to being a mechanism of resistance to EGFR‐targeted therapies such as cetuximab and panitumumab;^26^, ^27^, ^28^ however, because the used platform do not allow amplification assessment and budget reasons, we could not include *HER2* amplification analysis in this work.

The aim of this study was to characterize the underlying molecular changes associated with CRC in the Paraguayan population. For this purpose, we evaluated the histopathological features, the presence of common somatic pathogenic variants, and the MMR proteins status in a cohort of prospectively recruited Paraguayan patients, in order to incorporate these determinations in the Paraguayan Health Care System to guide therapeutics decisions.

## 2 MATERIALS AND METHODS

### 2.1 Patient selection and data collection

The design of the study was exploratory and prospective. A total of 36 consecutive and nonrelated CRC patients were recruited from May 2017 to February 2018 at the Medical Oncology Units from two public hospitals in Paraguay: Hospital de Clinicas and Instituto Nacional del Cancer (INCAN). Patient eligibility criteria included clinical and histological diagnoses of advanced CRC chemo‐naive or in progression to a first‐line chemotherapy for the advanced disease, Eastern Cooperative Oncology Group Performance Status (ECOG) 0 or 1, and potential candidates to receive chemotherapy in combination with target therapies according to the clinical guidelines.^29^

Clinical and pathological information, including age, sex, tumor location, histological grade, and treatments were collected (Table 1). All study subjects gave written informed consent, and the study was approved by the Biomedical Research Institute INCLIVA and the Hospital de Clínicas‐Paraguay Ethics Committee.

**Table 1:** Clinical and pathological characteristics of patients diagnosed with colorectal cancer (CRC)

Mean age (range)	52 (20‐74)
Sex (%)	
Female	13 (36.1)
Males	23 (63.9)
Tumor location (%)	
Right	14 (39)
Left	22 (61)
Histology grade (1‐3)	
G1	1 (3)
G2	26 (72)
G3	9 (25)
Mutation profile (%)	
All RAS WT	16 (44.4)
RAS/BRAF mutated	15 (41.6)
Other alterations	5 (14)
MSS status (%)	
MSS	29 (80.5)
Microsatellite instability	5 (13.8)
Unknown	2 (5.7)
Familiar CRC/breast/ovarian (%)	7 (19.4)
Unknown	29 (80.6)
Clinical stage at diagnosis (%)	
Stage I‐III	6 (16.7)
Stage IV	30 (83.3)
First‐line treatments administered (%)	
5‐FU + oxaliplatin/irinotecan	27 (75)
5‐FU + oxaliplatin/irinotecan + Bevacizumab	9 (25)

Formalin‐fixed paraffin‐embedded (FFPE) tissues were evaluated for their tumor content, and sections containing more than 30% tumor cells were selected by a dedicated pathologist. Genomic DNA was isolated from four unstained sections of 20 μm and diluted to a final solution of 10 ng/μL. This was done using the QIAamp DNA FFPE tissue kit (QIAGEN). DNA concentration was quantified in samples by NanoDrop (NanoDrop Technologies, Wilmington, DE).

### 2.2 Immunohistochemistry

IHC assays were performed in the 36 CRC patients as we previously described.^30^ The primary antibodies used were MLH1 (clone IR079, dilution 1:100; Dako), MSH2 (clone IR085, dilution 1:100; Dako), PMS2 (clone IR087, dilution 1:100; Dako), and MSH6 (clone IR086, dilution 1:100; Dako).

Tumors were considered positive if they present only nuclear staining, with or without cytoplasmic staining. Peritumoral lymphocytes, stromal cells, and non‐neoplastic epithelial cells were used as internal control. Only the complete loss of nuclear staining with positive internal control was classified as loss of MMR protein expression and was considered as evidence of MSI. Normal expression was defined as the presence of nuclear staining in tumor cells, irrespective of the intensity.

### 2.3 Sequenom MassARRAY somatic mutation genotyping

The Sequenom MassARRAY and OncoCarta Panel v1.0 were used following the manufacturer's protocol (Sequenom, San Diego, CA; http://agenabio.com/oncocarta-panel) as previously described.^19^ The panel consisted of 24 multiplex assays capable of detecting 238 pathogenic variants in 19 oncogenes. This procedure was a rapid, cost‐effective method of identifying key cancer‐driving pathogenic variants across a large number of samples because it avoided complex bioinformatic analyses and assays were performed within 2 days. The amount of DNA added to the polymerase chain reaction was 20 ng per reaction. DNA was amplified using the OncoCarta PCR primer pools. Unincorporated nucleotides were inactivated by shrimp alkaline phosphatase, and a single‐base extension reaction was performed using extension primers that hybridize immediately adjacent to the mutations and a custom mixture of nucleotides. Salts were removed by the addition of a cation‐exchange resin. Multiplexed reactions were spotted onto SpectroCHIP II arrays, and DNA fragments were resolved by MALDI‐TOF on the Compact Mass Spectrometer (Sequenom). An additional customized panel was used for some of the samples as a quality control. Details regarding genes and hot‐spot pathogenic variants analyzed within the OncoCarta panel are provided within Table S1.

### 2.4 Statistical analyses

Statistical analyses were carried out by IBM SPSS v 20.0. A *P* value of <0.05 was considered statistically significant. Comparison between clinical and pathologic patient's characteristics was done using the chi‐squared test, the Fisher's exact test, or the Wilcoxon rank test for qualitative and quantitative variables, respectively, prior assessment of normality using the Shapiro‐Wilk test. Tumor‐specific survival (TSS) was calculated from the time of diagnosis to the time of death because of tumor‐related causes or until the last known follow‐up. Survival curves were performed using the Kaplan‐Meier analysis compared to the log‐rank test. Multivariate regression analysis was carried out using Cox proportional hazards models with stepwise selection, including those variables significantly correlated with the survival probability on the univariate analysis. SPSS v20.0 was used to analyze the results.

Genomic data were analyzed using the Sequenom MassARRAY Typer Analyzer 4.0 Software to visualize the mass spectra for pathogenic variants and to determine the frequency of mutant and wild‐type alleles. The lower thresholds for mutation detection have been reported between 5% and 10%.^31^ In order to reduce putative false positives, we set the threshold at 10%. More specifically, only mutations with frequencies higher than 10% were taken as positive results. Pathogenic variants were manually reviewed by use of visual and raw spectrum patterns. Two different personnel in the laboratory scored pathogenic variants, and no discrepancies were observed. Analyses were performed using IBM SPSS Statistics for Windows, Version 20.0. Armonk, NY: IBM Corp (IBM Corp. Released 2010).

Pathogenic variant Waterfall plot of the patients’ dataset was performed through visualization of the plot by cBioportal‐OncoPrimer v1.18.0 (www.cbioportal.org/OncoPrimer) and Lolliplots have been draw with cBioportal‐Mutation Mapper v1.18.0 (www.cbioportal.org/MutationMapper).^32^, ^33^

All the molecular alterations found in our series were classified according to OncoKB classification:

OncoKB classification OncoKB is a precision oncology knowledge base and contains information about the effects and treatment implications of specific cancer gene alterations. Treatment information is classified using the Levels of Evidence system which assigns the clinical actionability (ranging from standard‐of‐care to investigational or hypothetical treatments) to individual mutational events. OncoKB currently contains treatment information for Level 1 and Level 2 (those alterations which are FDA‐recognized or considered standard care biomarkers predictive of response to FDA‐approved drugs in specific disease settings), Level 3 alterations (those alterations which are considered predictive of response based on promising clinical data to targeted agents being tested in clinical trials), and Level 4 (those alterations which are considered predictive of response based on compelling biological evidence to targeted agents being tested in clinical trials).^34^ OncoKB classification was used to analyze the results according to their clinical interest as targetable alterations or as biomarkers of resistance.

## 3 RESULTS

### 3.1 Patient characteristics

**Figure 1:**
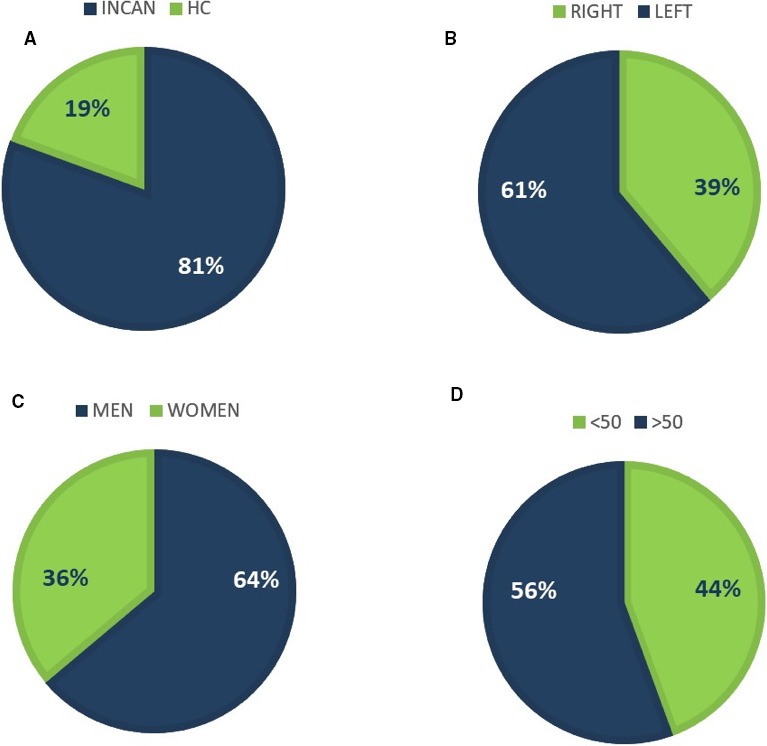
Demographic characteristics of the series. A, Percentage of patients recruited from the two different participating centers: INCAN, Instituto Nacional del Cáncer; HC, Hospital de Clínicas, (B) percentage of patients with right (green) and left (blue) location of tumor lesions, (C) distribution of men and women among the samples analyzed, (D) distribution of patients according to the age at the moment of diagnosis > or <50 years old

Seven patients (19%) from Hospital de Clínicas and 29 patients (81%) from INCAN were included in the study . The mean age of the patients was 52 years with a range from 20 to 74 years. Twenty‐three patients were males (63.9%) and 13 were females (36.1%). Eighty‐three percent of the patients included in the study have advanced‐stage tumors at the moment of the diagnosis with more tumors located in the left (61%, 22 cases) than in the right side (39%, 14 patients) (all demographic characteristicas can be seen in Figure 1). All the patients received a first‐line chemotherapy. In addition, nine of the patients received bevacizumab treatment.

Clinical characteristics of the patients are shown in Tables 1 and S2.

### 3.2 Mutational analysis

The molecular characterization analysis showed 16 patients (44.4%) wild‐type for all the oncogene regions analyzed with the Oncocarta panel including KRAS wild‐type (Level 1 OncoKB classification). We have been able to identify 32 hot‐spot mutations on seven oncogenes among 20 patients (55.6%). A total of 16 different oncogenic mutations were identified. Considering that the threshold of mutation detection with the technology applied is 10%, we observed a median average mutation load of 22.44% among all the samples, ranging from 8.5% up to 53.7%.

**Figure 2:**
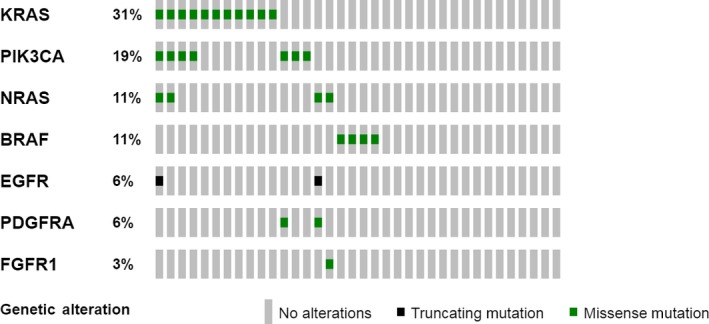
Mutational Waterfall plot of the patients’ dataset. Data have been obtained by analyzing the Oncocarta^™^ v1.0 panel (MassARRAY^R^ System by Agena Bioscience^™^). Visualization of the plot by cBioportal‐OncoPrimer v1.18.0^32^, ^33^ (www.cbioportal.org/OncoPrimer). Colored squares mean the type of alteration detected: green indicates missense mutation, whereas black identifies truncating mutation. All grey squares identify one patient; when they are without any other color means that no alterations are present in the sample

**Figure 3:**
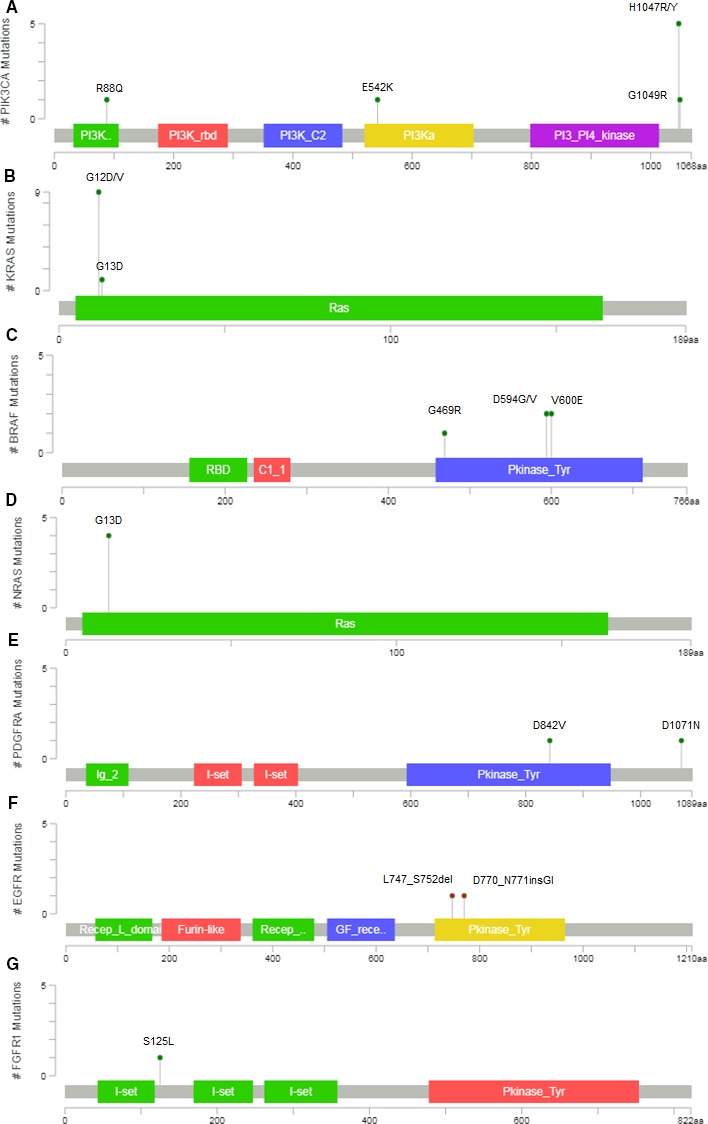
Mapping of Mutations detected in seven oncogenes. Lolliplots have been draw with cBioportal‐Mutation Mapper v1.18.0 (www.cbioportal.org/MutationMapper).^32^, ^33^ The plot identifies the different domains in each respective protein. The nature of the mutations and its position is shown. The number of times each mutation has been detected is shown with the left scale and is represented by the height of dot. A, *KRAS*, (B) *PIK3CA*, (C) *NRAS*, (D) *BRAF*, (E) *EFGR,* (F) *PDGFRA,* and (G) *FGFR1*

The most frequently mutated genes were *KRAS* in 11 tumors (seven with p.G12D, three with p.G12V, and one with p.G13D) (level R1 OncoKB classification), *PIK3CA* in eight tumors (five mutations in the hot‐spot p.H1047R/Y, one in p.G.1049R, p.E542K, and p.R88Q, respectively), *NRAS* in four tumors (all in p.G13D) (level R1 OncoKB classification), and *BRAF* in four tumors (two in p.V600E [level 3 OncoKB classification], one in p.D594V, and one in p.G469R) . Seven out of 20 patients have two or more mutations. Four patients have co‐occurrence mutations in *KRAS* and *PIK3CA*. Strikingly, two of the *KRAS/PIK3CA* mutated tumors carried also another mutation in *NRAS* (p.G13D). Low frequently mutated genes were *EGFR*,* PDGFRA*, and *FGFR1* and variations in these genes appeared in co‐occurrence with mutations in the most frequently mutated genes mentioned above (see Figure 2). Plots with number of patients with mutations, frequency of mutated genes, and co‐occurrences are presented in Figure 2. Full details of protein products of the mutated genes, specific mutations detected, its localization in protein domain, and their frequency are presented in Figure 3.

### 3.3 IHC of mismatch repair (MMR) proteins

**Figure 4:**
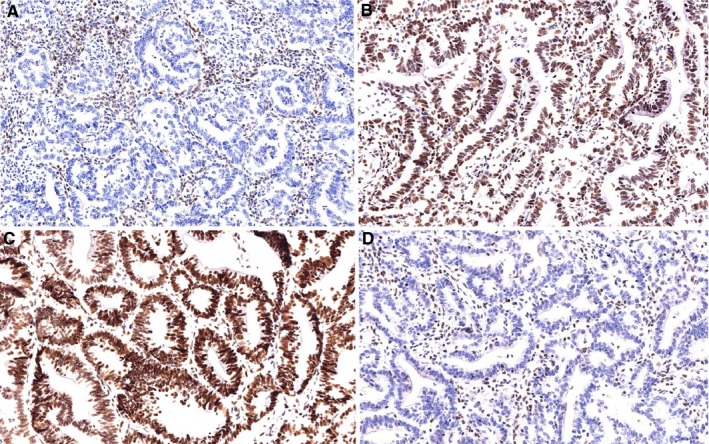
Immunohistochemistry study of mismatch repair proteins expression. Complete loss of nuclear staining for MSH6 and MSH2 in tumor cells, with positive internal control in stromal lymphocytes and fibroblasts (A: MSH6 40×, D: MSH2 40×) Retained MLH1 and PMS2 nuclear expression in tumor cells (B: MLH1 40×, C: PMS2 40×)

Five patients (14%) presented MSI. Three of them were younger than 50 years old and had a family history of CRC. The other two MSI cases were 54 and 57 years old without any family history of cancer. Two patients presented lost of MSH2 and MSH6 expression (a 27 years old female and 41 years old male). Clinical, pathological, and molecular characteristics of patients with MMR protein expression alterations are shown in Table 2 and Figure 4.

**Table 2:** Clinical, pathological, and molecular characteristics of patients with mismatch repair (MMR) protein expression alterations

Gender	Age	Location	Molecular profile	MMR protein expression (immunohistochemistry)	Family history (Bethesda or Amsterdam criteria)
Female	57	Right	BRAF (11.0%)	PMS2/MLH1 lost	−
Female	27	Left	KRAS (25.7%)	PMS2/MLH1 lost	+
Female	27	Left	KRAS (29.0%)	MSH6 lost and MSH2 heterogeneous expression	+
Male	41	Left	WT	MSH2/MSH6 lost	+
Male	54	Right	WT	PMS2/MLH1 lost	Unknown

### 3.4 Clinical correlations of patients and survival data

There was a significant correlation between tumor location (right vs left) and age (> or <50 years old) (*P* < .0.05). All cases of tumors located in the right colon were patients >50 years old. No other correlations between the clinical characteristics (gender, age, MSS status, or mutation profile) were found significant.

**Figure 5:**
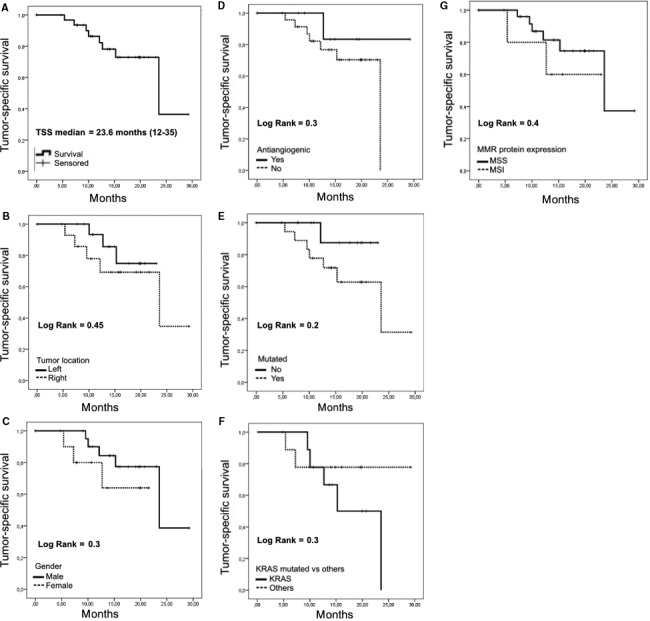
Kaplan‐Meier curves of tumor‐specific survival (TSS) of the colorectal cancer patients. A, Tumor‐specific survival of the all series. B, Tumor‐specific survival according to the tumor location (left vs right). C, Tumor‐specific survival according to the gender (males vs females). D, Tumor‐specific survival according to the treatment administered (chemotherapy + antiangiogenics vs chemotherapy alone). E, Tumor‐specific survival according to the mutation profile (mutated/no mutated). F, Tumor‐specific survival according to the mutation profile: KRAS vs other mutations

The mean TSS at the moment of the analysis was 23.6 months (12‐35 months). No differences were found in TSS according to the mutational status, gender, MSS status, or the treatment administered (chemotherapy ± bevacizumab). Survival curves are represented in Figure 5. However, a better survival trend to signification can be observed in relation with the following clinicopathological characteristics: patients without any mutation (candidates to anti‐EGFR therapies), male patients, left‐side colon tumors, patients treated with antiangiogenics, and patients with preserved MMR protein expression.

IHC and molecular profile analysis provided relevant information for a personalized medicine approach for all the cases. In our series, 45% of the patients had *RAS* wild‐type tumors that could benefit from anti‐EGFR therapies. Moreover, five patients with MSI profile could benefit from immunotherapy with checkpoint inhibitors such as pembrolizumab or nivolumab.^25^, ^35^

## 4 DISCUSSION

CRC incidence rates vary widely, with eightfold and sixfold variations by world regions for colon and rectal cancer, respectively. Therefore, CRC could be considered a marker of socioeconomic development, as is seen in countries undergoing a major development transition, where incidence rates tend to rise uniformly with the increasing Human Development Index.^1^ These rises in incidence—particularly the generational changes detected in most age‐period‐cohort analyses—point to the influence of dietary patterns, obesity, and lifestyle factors. However, mortality rates are declining in more developed countries due to improvements in survival through the adoption of best practices in cancer prevention, early diagnosis through screening approaches, and personalized treatments.^36^ Actually, molecular characterization has become a useful and mandatory tool for a personalized medicine approach in CRC;^10^ however, screening programs are not available in all developing countries.

The situation in Paraguay is alarming, with a CRC incidence rising during the last 20 years for both sexes from a population rate/100.000 of 3.66 and 2.87 for males and females, respectively, in 1998 to 5.51 for males and 4.88 for females in 2015.^1^ Thus, there is an urgent need for the implementation of effective strategies at primary, secondary, and third levels of prevention that could improve the results in Paraguay. For secondary prevention, the first steps have been made during 2018 with the implementation of CRC screening; however, it is still in a very initial stage. Regarding patients with advanced CRC, targeted therapies associated with chemotherapy improve the outcomes.^12^, ^37^ Nevertheless, their high costs limit their availability and use in Paraguay. Therefore, precision medicine through molecular testing is needed due to identify those patient candidates for targeted therapies and for the budgetary calculations of the National Health Plans.

The aim of our study was to characterize the underlying molecular changes associated with CRC in the Paraguayan population through MassARRAY technology, in order to incorporate these determinations into the Paraguayan Health Care System to guide therapeutics decisions.

Although the limited number of patients included, our work is the first published data of advanced CRC in Paraguay. We found 45% of patients who could benefit from anti‐EGFR therapies according to their mutational profile (*RAS* wild‐type). Patients with CRC being considered for anti‐EGFR therapy must receive *RAS* mutational testing. Based on the American Society for Clinical Pathology, College of American Pathologists, Association for Molecular Pathology, and American Society of Clinical Oncology Consensus, mutational analysis should include *KRAS* and *NRAS* codons 12 and 13 of exon 2, 59 and 61 of exon 3, and 117 and 146 of exon 4 (“expanded” or “extended” *RAS*)^38^ Moreover, this Consensus recommend *BRAF* p.V600 position mutational analysis in CRC tissue in selected patients with colorectal carcinoma for prognostic stratification, and also in dMMR tumors with loss of *MLH1* to evaluate for Lynch syndrome risk. Presence of a *BRAF* mutation strongly favors a sporadic pathogenesis. 38 Although, more than 80% of the patients recruited were diagnosed with advanced disease, we detected just above 55% of them with oncogenic mutations. From the 19 oncogenes evaluated, only seven had mutations (including *KRAS*,* NRAS*,* BRAF*,* PI3KCA*,* PDGFRA*,* EGFR*, and *FGFR*), which are in line with the data reported in the COSMIC database and in previous studies. Nevertheless, we found some differences, although we cannot draw conclusions due to the limited number of cases of our study, *BRAF* activating mutations occur in about 8% of advanced disease patients with CRC in most of the series^39^comparing with our results that showed 11% of BRAF mutations. Another interesting study remarks how NRAS defines a molecular subset with distinct clinical characteristics from KRAS‐mutant and wild‐type mCRC. The study by Cercek et al. described NRAS mutations enriched in left‐sided primary tumors and among African Americans conferring a poor survival and worse outcomes than either KRAS‐mutant or wild‐type mCRC.^40^ Interestingly in our study, all NRAS mutations were accompanied by other co‐mutations (Table S2). In contrast with these results, in our series, three of the cases with NRAS mutations were found in patients with CRC diagnosed in the right colon and only one in the left side.^40^

The largest CRC series of patients analyzed by MassARRAY Oncocarta^™^ Panel included 239, 254, and 2299 patients.^20^, ^31^, ^41^, ^42^ In a previous study from our group,^19^ mutations were detected in 48 out of 75 CRC cases (64.2%) using this technology. Specifically, mutations were found mainly in the *KRAS*,* PIK3CA*, and *KIT* genes. Although alterations, such as the amplification of *HER2*, NTRK fusions, POLE mutations, and others of interest are not possible to test through Oncocarta panel, this platform covers the hot‐spots spectrum for the clinical decisions on anti‐EGFR therapies.

In our experience, the MassARRAY technology in combination with the OncoCarta Panel successfully detected frequent cancer mutations in degraded DNA isolated from FFPE samples and covers up to 95% of known druggable markers. Thus, it provides an efficient mutation screening for clinical research trials and with high concordance with NGS technologies. Our results confirmed that MassARRAY technology is a rapid and effective method for identifying key cancer‐driving mutations across a large number of samples, which allows for a more appropriate selection for personalized therapies, and could be a cost‐effective method for the molecular profiling in Paraguay.

MSI in mCRC has a global frequency of 4%. As mentioned before, the analysis of MMR is relevant for the diagnosis of hereditary syndromes, as well as for the identification of biomarkers that would guide immunotherapy with immune checkpoint blockade inhibitors.^43^ The analysis of MMR can be done through IHC and PCR techniques, both methods are available in Paraguay. Validated IHC detection of MMR proteins is a feasible and cost‐effective method for MSI identification in CRC. Although normal immunoreactivity can be seen in up to 10% of MSI cases therefore, MSI DNA testing may be performed.^38^

Recently, checkpoint inhibitors have been included into the national drugs bank. In our series, we detected MSI in five cases, two of them showing loss of MSH2 and MSH6, a pattern highly suggestive of Lynch syndrome. Those cases should be comprehensively analyzed in genetic counseling units, in order to evaluate the presence of germline mutations. In our series, the young average age of presentation (just over 50 years), and the presence of MMR proteins loss of MSH2 and MSH6 in two cases (5%) highlights the importance of the urgent implementation of genetic counseling units in Paraguay. MMR genetic analysis at germline level is strongly recommended for those cases younger than 50 years old with a family history of CRC.

Despite the low number of patients included in the study, we were able to draw the mutation profile of CRC patients in Paraguay. In addition, the study would provide relevant clinical and molecular information to be included in Public Oncology Reference Hospitals of Paraguay, as well as the usefulness of Sequenom MassARRAY technology for the molecular profiling and the MSI testing to guide the therapeutics to guide the treatment of advanced CRC disease.
