# Supplementary material for: Molecular profile in Paraguayan colorectal cancer patients, towards to a precision medicine strategy
Source: Cancer Med. 2019 May 6;8(6):3120–30. doi: 10.1002/cam4.2191 (PMC6558499; doi:10.1002/cam4.2191)
Supplement: Supplementary file 1 [file CAM4-8-3120-s001.doc]

List of mutations analysed using the Sequenom MassARRAY Technology.

| **ABL1** | G250E, Q252H, Y253H/F, E255K/V, D276G, F311L, T315I, F317L,M351T, E355G, F359V, H396R |
| --- | --- |
| **AKT1** | E17del, Q43*, V167A, E319G, L357T, P388T, V461L |
| **AKT2** | S302G, R371H |
| **BRAF** | G464R/V/E, G466R, F468C, G469S/E/A/V/R, D594V/G, F595L, G596R, L597S/R/Q/V, T599I, V600E/K/R/L, K601N/E |
| **CDK4** | R24C/H |
| **EGFR** | R108K, T263P, A289V, G598V, E709K/H, E709A/G/V, G719S/C/A, M766_A767insAI, S768I, V769_D770insASV, V769_D770insCV, D770_N771>AGG/V769_D770insASV, D770_N771insG, N771_P772>SVDNR, P772_H773insV, H773>NPY, H773_V774insNPH/PH/H, V774_C775insHV, T790M, L858R, L861Q, E746_T751del, E746_A750del, S752D, L747_E749del, L747_T750del, L747_T751del, L747_S752del, P753S, A750P, T751A, T751P, T751I, S752I/F, S752_I759del, L747_Q ins, E746_T751del, I ins (combined), E746_A750del, T751A (combined), L747_E749del, A750P (combined), L747_T750del, P ins (combined), L747_S752del, Q ins (combined) |
| **ERBB2** | L755P, A775_G776insYVMA, G776S/LC, G776VC/VC, S779_P780insVGS, P780_Y781insGSP |
| **FGFR1** | S125L, P252T |
| **FGFR3** | G370C, Y373C, A391E, K650Q/E/T/M |
| **FLT3** | D835H/Y, I836del |
| **HRAS** | G12V/D, G13C/R/S, Q61H/L/R/P/K |
| **JAK2** | V617F |
| **KIT** | D52N, Y503_F504insAY, K550_K558del, P551_V555del, M552L, Y553_Q556del, W557R/R/G, K558_V560del, K558_E562del, V559D/A/G/I, V559del, V559_V560del, V560D/G, V560del, E561K, Y568D, Y570_L576del, L576P, D579del, F584S, P585P, K642E, D816V/H/Y, V825A, E839K |
| **KRAS** | G12V/A/D/C/S/R/F, G13V/D, A59T, Q61E/K/L/R/P/H |
| **MET** | R970C, T992I, Y1230C, Y1235D, M1250T |
| **NRAS** | G12R/S/C/V/A/D, G13R/S/C/V/A/D, A18T, Q61E/H/K/L/P/R |
| **PDGFRA** | V561D, S566_E571>K, T674I, F808L, D842V, D842_H845del, I843_D846del, I843_S847>T, D846Y, N870S, D1071N |
| **PIK3CA** | R38H, R88Q, N345K, C420R, P539R, E542K, E545K, Q546K, H701P, C901F, M1043I, H1047R/L/Y |
| **RET** | E632_L633del, C634R/W/Y, A664D, M918T |

Sequenom assays, perform at the Biomedical Research Institute – INCLIVA, rapidly confirm hotspot mutations in key oncogenes with the highly robust MassARRAY® System. Each assay is based in an extension of a specific probe.

A ready-to-use panel for 238 mutations in 19 oncogenes (Oncocarta^TM^ version 1.0) designed by the Sequenom Company was employed.
