# Supplementary material for: Molecular profile in Paraguayan colorectal cancer patients, towards to a precision medicine strategy
Source: Cancer Med. 2019 May 6;8(6):3120–30. doi: 10.1002/cam4.2191 (PMC6558499; doi:10.1002/cam4.2191)
Supplement: Supplementary file 2 [file CAM4-8-3120-s002.doc]

| **Table supplementary 2: Clinical and Molecular characteristics of patients.** | | | | | | | | |
| --- | --- | --- | --- | --- | --- | --- | --- | --- |
| **LAB CODE** | **CENTRE** | **GENDER** | **AGE YO** | **TUMOR LOCATION** | **GENE** | **MUT** | **%** | **MMR EXPRESSION** |
| MTCYD1 | INCAN | Male | < 50 | Right |  | ALL WT |  | Normal |
| MT-CYD2 | INCAN | Female | < 50 | Left | KRAS | G12D | 29,50% | MSH6 & MSH2 (-) |
| MT-CYD3 | INCAN | Female | ≥50 | Left | BRAF | V600E | 17,30% | Normal |
| MT-CYD4 | INCAN | Male | < 50 | Left | PIK3CA | G1049R | 11.00% | NV |
| MT-CYD5 | INCAN | Female | < 50 | Left | KRAS | G12D | 25,70% | PMS2 & MLH1(-) |
| MT-CYD6 | HC | Male | ≥50 | Left | KRAS | G12V | 31.20% | Normal |
| MT-CYD7 | HC | Female | < 50 | Left | KRAS | G13D | 25,60% | Normal |
| MT-CYD8 | HC | Male | < 50 | Left | KRAS | G12D | 24,20% | Normal |
| MT-CYD9 | INCAN | Male | < 50 | Right |  | ALL WT |  | Normal |
| MT-CYD10 | INCAN | Male | < 50 | Left | BRAF | V600E | 18,80% | Normal |
| MT-CYD11 | INCAN | Male | < 50 | Left |  | ALL WT |  | Normal |
| MT-CYD12 | HC | Female | ≥50 | Right | BRAF | D594V|G | 11,20% | PMS2 & MLH1 (-) |
| MT-CYD13 | HC | Male | ≥50 | Right | NRAS | G13D | 25,60% | Normal |
|  |  |  |  |  | PIK3CA | H1047R | 11,80% |  |
|  |  |  |  |  | KRAS | G12D | 26,80% |  |
| MT-CYD14 | HC | Female | ≥50 | Right | FGFR1 | S125L | 42,50% | Normal |
|  |  |  |  |  | NRAS | G13D | 25,90% |  |
| MT-CYD15 | HC | Male | < 50 | Right | KRAS | G12V | 16,40% | Normal |
| MT-CYD16 | INCAN | Male | ≥50 | Right |  | ALL WT |  | Normal |
| MT-CYD17 | INCAN | Female | ≥50 | Left |  | ALL WT |  | Normal |
| MT-CYD18 | INCAN | Female | < 50 | Left | KRAS | G12D | 28,10% | NV |
|  |  |  |  |  | PIK3CA | H1047R | 15,10% |  |
| MT-CYD19 | INCAN | Male | < 50 | Left | BRAF | G469R | 24,90% | Normal |
| MT-CYD20 | INCAN | Male | ≥50 | Left |  | ALL WT |  | Normal |
| MT-CYD21 | INCAN | Female | ≥50 | Left | PIK3CA | R88Q | 39,50% | Normal |
|  |  |  |  |  | PIK3CA | H1047Y | 13,60% |  |
|  |  |  |  |  | PDGFRA | D1071N | 10,10% |  |
| MT-CYD22 | INCAN | Female | ≥50 | Right |  | ALL WT |  | Normal |
| MT-CYD23 | INCAN | Male | ≥50 | Right | EGFR | D770_N771insG | 33,80% | Normal |
|  |  |  |  |  | NRAS | G13D | 10,40% |  |
|  |  |  |  |  | PDGFRA | D842V | 53,70% |  |
| MT-CYD24 | INCAN | Female | ≥50 | Right |  | ALL WT |  | Normal |
| MT-CYD25 | INCAN | Female | < 50 | Left | PIK3CA | H1047R | 11,10% | Normal |
|  |  |  |  |  | KRAS | G12V | 8,50% |  |
|  |  |  |  |  | NRAS | G13D | 9,50% |  |
|  |  |  |  |  | EGFR | L747_S752del, P753S | 11,20% |  |
| MT-CYD26 | INCAN | Male | < 50 | Left |  | ALLWT |  | MSH2 &MSH6(-) |
| MT-CYD27 | INCAN | Male | ≥50 | Left | KRAS | G12D | 34,00% | Normal |
|  |  |  |  |  | PIK3CA | E542K | 15,30% |  |
| MT-CYD28 | INCAN | Male | ≥50 | Right | PIK3CA | H1047Y | 11,70% | Normal |
| MT-CYD29 | INCAN | Male | ≥50 | Right |  | ALL WT |  | PMS2 & MLH1(-) |
| MT-CYD30 | INCAN | Male | ≥50 | Left |  | ALL WT |  | Normal |
| MT-CYD31 | INCAN | Male | ≥50 | Right | KRAS | G12D | 41,50% | Normal |
| MT-CYD32 | INCAN | Male | ≥50 | Left |  | ALL WT |  | Normal |
| MT-CYD33 | INCAN | Male | ≥50 | Right |  | ALL WT |  | Normal |
| MT-CYD34 | INCAN | Female | < 50 | Left |  | ALL WT |  | Normal |
| MT-CYD35 | INCAN | Male | < 50 | Left |  | ALL WT |  | Normal |
| MT-CYD36 | INCAN | Male | ≥50 | Left |  | ALL WT |  | Normal |

INCAN: Instituto Nacional del Cancer Capiatá-Paraguay; HC Hospital de Clínicas. San Lorenzo-Paraguay; WT: Wide type; NV: not valuable.
